# Improved algorithms for approximate string matching (extended abstract)

**DOI:** 10.1186/1471-2105-10-S1-S10

**Published:** 2009-01-30

**Authors:** Dimitris Papamichail, Georgios Papamichail

**Affiliations:** 1 Department of Computer Science, University of Miami, Coral Gables, Miami, USA; 2 National Center of Public Administration, Athens, Greece

## Abstract

**Background:**

The problem of approximate string matching is important in many different areas such as computational biology, text processing and pattern recognition. A great effort has been made to design efficient algorithms addressing several variants of the problem, including comparison of two strings, approximate pattern identification in a string or calculation of the longest common subsequence that two strings share.

**Results:**

We designed an output sensitive algorithm solving the edit distance problem between two strings of lengths *n *and *m *respectively in time O((*s *- |*n *- *m*|)·min(*m*, *n*, *s*) + *m *+ *n*) and linear space, where s is the edit distance between the two strings. This worst-case time bound sets the quadratic factor of the algorithm independent of the longest string length and improves existing theoretical bounds for this problem. The implementation of our algorithm also excels in practice, especially in cases where the two strings compared differ significantly in length.

**Conclusion:**

We have provided the design, analysis and implementation of a new algorithm for calculating the edit distance of two strings with both theoretical and practical implications. Source code of our algorithm is available online.

## Background

Approximate string matching is a fundamental, challenging problem in Computer Science, often requiring a large amount of computational resources. It finds applications in different areas such as computational biology, text processing, pattern recognition and signal processing. For these reasons, fast practical algorithms for approximate string matching are in high demand. There are several variants of the approximate string matching problem, including the problem of finding a pattern in a text allowing a limited number of errors and the problem of finding the number of edit operations that can transform one string to another. We are interested in the latter form in this paper.

The edit distance *D*(*A, B*) between two strings *A *and *B *is defined in general as the minimum cost of any sequence of edit operations that edits *A *into *B *or vice versa. In this work we will focus on the Levenshtein edit distance [1], where the allowed edit operations are insertion, deletion or substitution of a single character, with each operation carrying a cost of 1. The distance measure which uses this type of operation is often called the unit-cost edit distance and is considered the most common form. The weighted edit distance allows the same operations as the Levenshtein edit distance, but each operation may have an arbitrary cost.

In the literature there exist a number of algorithms dealing with the calculation of the edit distance between two strings. The basic dynamic programming algorithm that solves the problem in *O*(*mn*) time and linear space has been invented and analyzed several times in different contexts [2-7], published between 1968 and 1975. Early on there was an algorithm by Masek and Paterson [8], building on a technique called the "Four-Russian paradigm" [9], which computes the edit distance of two strings over a finite alphabet in time *O*(*mn*/log^2 ^*n*). This algorithm is not applicable in practice, since it can outperform the basic algorithm only then the input size is exceeding 40 GB. All these algorithms can also be used to calculate the *alignment *of two strings, in addition to their edit distance. A modification of the basic algorithm by Hirschberg [10] allows the alignment calculation to be performed using linear space as well.

A few years later in 1985, Ukkonen arrived at an *O*(*s*·min(*m*, *n*)) time algorithm, using space *O*(min(*m*, *n*, *s*)) [11], where s is the edit distance of the two strings compared, creating a very efficient output sensitive algorithm for this problem. The following year, Myers published an algorithm for the Longest Common Substring (*LCS*) problem, which is similar to the edit distance problem, which has *O*(*s*^2 ^+ (*m *+ *n*) log(*m *+ *n*)) time and linear space complexity [12]. In achieving this result, a generalized suffix tree of the input strings, supplemented by *Lowest Common Ancestor *(*LCA*) information, has to be used, which renders the solution impractical and only of theoretical value. The practical version of that algorithm needs *O*(*s*(*m *+ *n*)) time. On the other hand, a variation of Ukkonen's algorithm using *O*(*s*·min(*s*, *m, n*)) space leads to an efficient, straightforward implementation, using recursion. Lastly, the basic algorithm, although theoretically inferior, is the most commonly used, owing to its adaptability, ease of implementation, instruction value, and speed, the latter being a result of small constant factors.

In this paper we will present an O((*s *- |*n *- *m*|)·min(*m*, *n*, *s*) + *m *+ *n*) time and linear space algorithm to calculate the edit distance of two strings, which improves on all previous results, the implementation of which is practical and competitive to the fastest algorithms available. The quadratic factor in the time complexity now becomes independent of the longest string, with the algorithm performing its best when the two strings compared differ significantly in size.

## Methods

### Definitions

In this section we closely follow the notation and definitions in [11]. Let *A *= *a*_1_*a*_2_...*a*_*n *_and B = *b*_1_*b*_2_...*b*_*m *_be two strings of lengths *n *and *m *respectively, over a finite alphabet Σ. Without loss of generality, let *n *= *m*.

The edit operations defined in the previous section can be generalized to have non-negative costs, but for the sake of simplicity in the analysis of our algorithm we will concern ourselves only with the Levenshtein edit distance. We also assume that there is always an editing sequence with cost *D*(*A*, *B*) converting *A *into *B *such that if a cell is deleted, inserted or changed, it is not modified again. Under these assumptions the edit distance is symmetric and it holds 0 ≤ *s *≤ *max*(*n*, *m*). Since *n *≥ *m *and there is a minimum number of *n *- *m *insertions that need to be applied in transforming *A *into *B*, the last equation becomes *n *- *m *≤ *s *≤ *n*. The insertion and deletion operations are symmetric, since an insertion, when transforming *A *to *B*, is equivalent to a deletion in the opposite transformation, and vice versa. Both operations will be referred to as indels.

The basic dynamic programming algorithm employed to solve the edit distance problem, invented in a number of different contexts [2-7], makes use of the edit graph, an (*n *+ 1) × (*m *+ 1) matrix (*d*_*ij*_) that is computed from the recurrence:

$$\begin{array}{llll} d_{00} & = & 0 & \\ d_{ij} & = & \min( & d_{i-1,j-1}+(\text{if }a_{i}=b_{j}\text{ then }0\text{ else }1),\\ & & & d_{i-1,j}+1,\\ & & & d_{i,j-1}+1),i>0\text{ or }j>0. \end{array}$$

**Figure 1:**
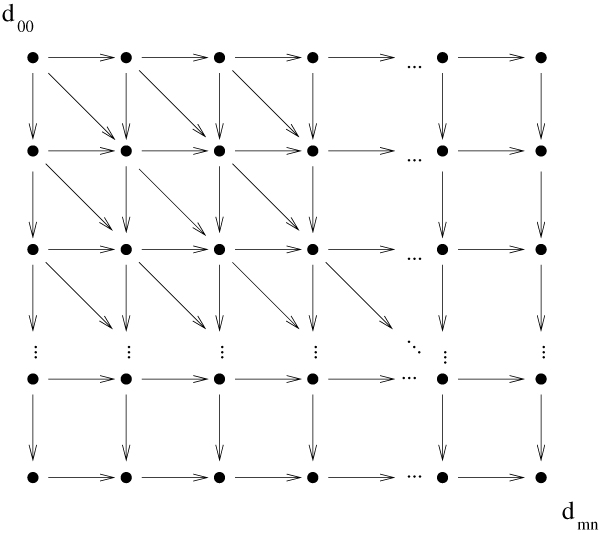
**Dependency graph**.

This matrix can be evaluated starting from *d*_00 _and proceeding row-by-row or column-by-column. This process takes time and space *O*(*mn*) and produces the edit distance of the strings in position *d*_*mn*_. The cells of the matrix (nodes of the graph) have dependencies based on this recurrence, forming the *dependency *or *edit graph*, a directed acyclic graph that is shown in Fig. 1. All edit graph nodes will be referred to as cells and all graph edges (edit operations) will be referred to as *transitions*.

To refer to the diagonals of (*d*_*ij*_) we number them with integers -*m*, -*m *+ 1, ..., 0, 1, ..., *n *such that the diagonal denoted by *k *consists of those *d*_*ij *_cells for which *j *- *i *= *k*. The diagonal *n *- *m*, where the final value *d*_*mn *_resides, is special for our purposes and we will call it main diagonal. The matrix cells between diagonals 0 and *n *- *m *(inclusive) consist the center of the edit graph/matrix, the lower left triangle between diagonals -1 to -*m *will be called the left *corner *of the graph and upper right triangle between diagonals *n *- *m *+ 1 and *n *will be called the *right corner *of the graph.

A *path *in the edit graph is a series of transitions connecting cells, similar to a path in a directed graph. Whenever we generally refer to a path, we will assume that the final cell it reaches is *d*_*mn*_. The optimal path will be a path originating at *d*_00_, and for which the sum of the costs of its transitions is minimal among all paths from *d*_00_.

### The concept

The basic dynamic programming algorithm evaluates unnecessary values of (*d*_*ij*_). This fact led Ukkonen [11] design an algorithm that is diagonal-based and computes cell values only between the diagonals -* s *and *n *- *m *+ *s*. He also observed that *d*_*i *+ 1, *j*+1 _∈ {*d*_*i*, *j*_, *d*_*i*.*j *_+ 1} and therefore the values along a diagonal are non-decreasing.

Both Ukkonen [11], for calculating the edit distance, and Myers [12], for calculating the length of the *Longest Common Substring *of two strings, designed their algorithms with a common feature: The iterations in evaluating the edit graph cells were score based, as opposed to column or row based in the basic algorithm. In each step they would increase the edit distance D by 1, starting at 0, and evaluate all cells with values *d*_*ij *_≤ *D*, meaning cells reachable with edit distance D, often omitting cells not contributing to the next iteration, by considering transitions between cells where the values are incremented.

The algorithm we present here builds on all previous observations and the main iteration is score based as well. But we also make use of the following facts:

1. *n *- *m *indels are unavoidable.

2. Additional indels are unavoidable when the optimal path strays away from the main diagonal.

3. Certain cells do not contribute to the optimal path or their contribution is redundant.

Points 1 and 2 follow from the fact that an indel is required to move to the next diagonal. At least *n *- *m *indels are required on any path that first reaches the main diagonal, and every time the path strays from the main diagonal, it must return to it.

In order to address the third fact, we will introduce the concept of *dominance*. We will say that cell *d*_*ij *_dominates cell *d*_*kl *_if no path through *d*_*kl *_defines a better edit distance than the optimal path through *d*_*ij*_. This implies that *d*_*ij *_has an equal or better potential to belong to the optimal path (which defines *s*) than *d*_*kl*_, and thus the latter and its paths do not need to be considered further.

Some dominance relations between cells can be spotted easily. Let us consider all possible paths starting from d_00_. If a match exists between characters *a*_1 _and *b*_1 _(*a*_1 _= *b*_1_), then we do not need to consider indel transitions from *d*_00 _to *d*_10 _and *d*_01_. In that case actually, all cells *d*_0*k *_for 1 ≤ *k *≤ *n *and *d*_*k*0 _for 1 ≤ *k *≤ *m *are dominated by *d*_11_. Since *a*_1 _matches *b*_1_, cell *d*_11 _obtains the value of 0. Then all cells d1k, 2 ≤ *k *≤ *n *can obtain a value of *k *- 1 through a path traversing *d*_11_. Any path through *d*_01 _cannot result in a smaller value for cells *d*_1*k*_, 2 ≤ *k *≤ *n*, since cells *d*_0,*k*-1 _have the same value. In a similar manner, cells in the second column starting at the third line are dominated by d_11_. These arguments apply not only to d_00 _but to all *d*_*ij *_in general, proving the following:

**Lemma 1**. *A cell d*_*ij *_*is dominated by d*_*i*+1,*j*+1 _*if a*_*j *_= *b*_*i*_.

Let us now consider what happens when *a*_1 _≠ *b*_1_. In this case we can still find dominated cells in the second row and column, depending on the first matching character position in each. Let us assume that the first character in A matching *b*_1 _is al, 2 ≤ *l *≤ *n*. All cells *d*_1*k*_, 2 ≤ *k *≤ *l *- 1 are dominated by *d*_11_, for the same reasons that were described earlier. And a similar domination relation exists in the columns. Before we generalize the dominance relation with a theorem, we will introduce a new scoring scheme to take advantage of the indel unavoidability, which will create another optimization criterion, monotonicity in the rows and columns of certain parts in our graph. For the new scoring scheme and for the rest of the description of our algorithm, we will divide our matrix into two parts, separated by the main diagonal. The first part includes the center and the left corner of the matrix, where the second part includes the right corner of the matrix, together with the main diagonal (which is shared by both parts). The scoring scheme and the algorithm described further on will be analyzed on the part of the matrix left of the main diagonal, although all theory works symmetrically on the part right of the main diagonal, by substituting the rows with columns and vice versa.

**Figure 2:**
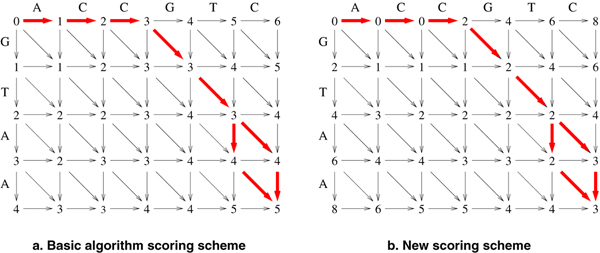
**Edit graphs under different scoring schemes**. Edit graph cell values and optimal paths under different scoring schemes.

The new scoring scheme, for the left part of the matrix, is implemented as follows: Every vertical transition (indel) incurs a cost of 2, since it strays away from the main diagonal and creates the need of another horizontal indel to compensate. All horizontal transitions do not carry any cost. The match and substitution costs remain 0 and 1 respectively. To obtain the edit distance s, we add *n *- *m *to the value of cell *d*_*mn*_. The transformation is illustrated through an example in Fig. 2.

To guarantee the correctness of an algorithm based on that scoring scheme, we will now prove the following lemma:

**Lemma 2**. *Under the new scoring scheme, the edit distance of A and B remains unchanged*.

*Proof*. It has already been shown that the edit distance is defined by an optimal path of the fewest possible edit operations carrying a cost, resulting in the minimum score at *d*_*mn*_. We will prove the following two statements:

1. The score obtained from the optimal path remains unchanged and

2. No other path can lead to a sequence of fewer edit operations and thus a smaller score/edit distance.

To prove the first statement, we note the following: The number of match and substitution transitions in the optimal path does not alter the edit distance in the new scoring scheme, since the costs of these operations have not changed. With the optimal path starting at diagonal 0 and ending at diagonal *n *- *m*, there are *n *- *m *indels which can be omitted from our calculation, since with the new scoring scheme we add these at the end. The only remaining edit operations to examine are vertical indels left of the main diagonal and horizontal indels right of the main diagonal, which must be accompanied by compensatory horizontal and vertical indels in the respective parts, or the optimal path cannot end up in the main diagonal. Since these indels come in pairs, with half of them carrying the cost of 2 and half the cost of 0 in the new scoring scheme, the final edit distance remains unchanged.

The second statement follows from the previous arguments, since any path under the new scoring scheme carries the same cost as before, so a new path with a better score than the previous optimal path score contradicts the optimality of the latter under the original scoring scheme.

Since with the new scoring scheme horizontal transitions do not carry a cost, the values of cells in every row in the left part of the matrix are monotonically decreasing. The same holds for the columns in the right part of the matrix, which leads to the following:

**Corollary 1**. *Under the new scoring scheme, the values of cells in rows left of the main diagonal and in columns right of the main diagonal are monotonically decreasing as the indices of the corresponding cells increase*.

Let us now consider all cells in a specific row *x*, left of the main diagonal. Values on this row are monotonically decreasing and we only need to keep the information of the first cells from the right where the values are changing (the leftmost cells of a series of cells with the same value), since the rest of the cells are dominated (can be reached with 0 cost from the aforementioned cells). Now, if we have two consecutive dominant cells *d*_*xy *_and *d*_*xz *_on row *x*, with *y *<* z *and *d*_*xy *_= *d*_*xz *_+ 1, then the value of *d*_*xy *_can be propagated through a transition to row *x *+ 1 only if a match exists between *b*_*x *_and *a*_*k*_, with *y *<* k *≤ *z*. In order to be able to locate such matches in constant time, we will create lookahead tables for each letter of the alphabet Σ, which can point to the next matching character from strings *A *and *B*. Basically these lookahead tables will be able to answer the question: Given a character *c *∈ Σ and a position 1 ≤ *k *≤ *n*, what is the smallest index *l *≥ *k *such that *a*_*l *_= *c*? And the same for string *B*. Such a lookahead table can be easily constructed in time and space O((*n *+ *m*)|Σ|), which for a fixed alphabet of constant size is linear, by traversing both strings in reverse order, once for each character of the alphabet.

One can easily verify that lemma 1 still holds, based on the same arguments used to prove it, under the new scoring scheme. In addition, the following corollary holds:

**Corollary 2**. *A cell d*_*ij *_*with value D dominates all cells d*_*i*-*k*, *j*-*k*_, 0 ≤ k ≤ max(*i, j*) *with values *≥ *D*.

*Proof*. It is easy to see, with a simple inductive argument, that a cell *d*_*ij *_dominates all parental cells on the same diagonal with the same score. Since any cell dominates itself with a higher score (because every path from that cell will have a higher score equal to the diffierence of the two scores), the corollary follows.   □

#### The algorithm

The algorithm works separately on the two parts of the matrix left and right of the main diagonal. The description of the algorithm considers only the part of the matrix lying left of the main diagonal, with the assumption that all operations are symmetric on the right part of the matrix. An exception occurs when we describe the interface between the two parts.

Our edit distance algorithm is score based. On each iteration the edit distance score is incremented by 1 and the part of the edit graph that can be reached with the current score is determined. The initial score is 0, although we should keep in mind that, since at the end we add *n *- *m *to the score – adjusting for the unavoidable indels that we get for free on horizontal transitions – it can be considered as if the score is initialized with the value *n *- *m*.

**Figure 3:**
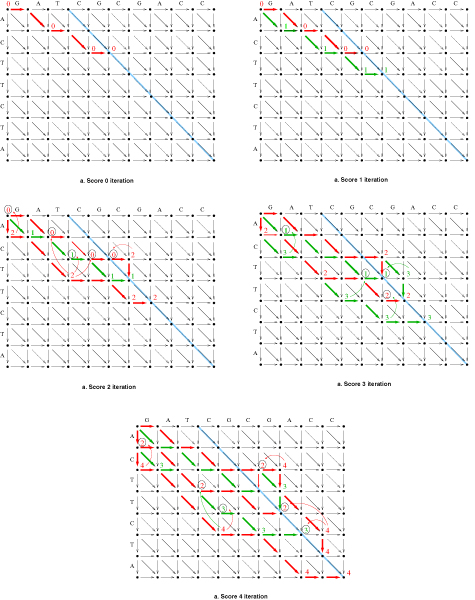
**Edit distance algorithm iterations**. Edit distance algorithm iterations. The main diagonal is depicted in blue, iteration transitions are drawn in red and green alternatively. Cells whose values are presented have been inserted in the list at the end of each iteration, where cells that their values are circled have been removed from the list, dominated by the cells they connect with arcs.

During each iteration, we store the values and positions of the cells we work with in a double linked list, which will be referred to simply as list. To store the position of a cell we actually need only the column index where the cell resides, for reasons that will be explained later. The initialization phase starts with the determination of the cells which can be reached with a score of 0. Since all horizontal and match diagonal transitions (diagonal transitions corresponding to matching characters) have a cost of 0, we follow horizontal transitions until we locate a match, then advance to the next line and repeat. The process ends when we reach the main diagonal. We do not need to keep information on all cells with 0 value, the first cell with a value of 0 on each line suffices, since all further cells are dominated. These dominant leftmost cells can be located in constant time for each line, by using the lookahead tables. When we encounter a series of matches on the same diagonal, we only need to keep the value of the last (bottom-right) cell, since all other cells are dominated. The indices of cells accessed through this process increase monotonically, as we advance forward through rows, columns and diagonals. The initialization finishes when the main diagonal is reached. Thus at the end of the initialization step we have a list of cells with 0 value, each of which resides on a different row, column and diagonal of the matrix. An example of the initialization phase can be found in Fig. 3a.

On each subsequent iteration of the algorithm and with each increasing value of the score, the linked list is updated with new cells that can be reached from members of the list. The algorithm at iteration *D*, with *D *also being the current score, starts from the top of the list and processes one cell at a time. For each list cell examined having a value of *D *- 1 or *D *- 2, as will be proved in lemma 3, we either follow a substitution transition, if the cell's value is *D *- 1 or a vertical indel transition if the cell's value is *D *- 2. Let's assume we are examining list cell *d*_*ij *_= *D *- 1. We know that *d*_*i*+1, *j*+1 _= *D*, since if *d*_*i*+1, *j*+1 _<* D *it would already be included in the list, unless dominated by another cell in the list, which is impossible since then *d*_*ij *_would in turn be dominated by *d*_*i*+1, *j*+1 _and would not be in the list during the current iteration. We now find the largest *k *for which *b*_*i*+*k *_= *a*_*j*+*k*_, *k *≥ 1 and insert cell *d*_*i*+*k*, *j*+*k *_in the list. That is the last cell in a series of match transitions, starting at *d*_*i*+1, *j*+1_, if any exist. Next, we examine the cells following *d*_*ij *_in the list and remove the ones that are dominated by *d*_*i*+*k*, *j*+*k*_. At this step, list cells *d*_*op *_in rows *o *<*i *+ *k *and on diagonals *o *- *p *such that *i *- *j *<*o *- *p *≤*n *- *m *are removed, all being dominated as proved later in theorem 1. Starting now at cell *d*_*i *+ *k*, *j *+ *k*_, we repeat the process performed in the initialization, with the difference that for each new cell inserted in the list, all subsequent cells in the list that are dominated by the new member are removed. This process will stop once the next identified match in the lookahead table falls inside the dominated area. Precisely, if *d*_*op *_is the last cell with value *D *that was inserted in the list, the next match from the lookahead tables resides at diagonal q and the next cell in the list resides at a diagonal *p *≤ *q *and row *r *≥ *o*, then the process of inserting new cells derived from *d*_*ij *_is terminated and we proceed to the next cell in the list.

Each iteration finishes once we reach the main diagonal. The reader can follow the procedure, through the five iterations in calculating the edit distance of strings *A *= 'GATCGCGACC' and *B *= 'ACTTCTA', in Fig. 3.

One special case that was not covered in the above description is the handling of a cell insertion following a vertical indel transition, when another dominated cell on the same diagonal exists in the list. In this case, the only position the dominated cell can occupy is previous to the current cell examined, from which the transition emanated. This results in the removal of the dominated cell. This special case only requires a constant number of operations and does not alter the complexity of the algorithm. As already mentioned, the part of the matrix right of the main diagonal is processed in a symmetric way. At the end of each iteration, the cells of the main diagonal, which belongs to both parts, have to be updated. These cells reside at the end of the lists for both parts and the update is performed in constant time as well.

We will now proceed to prove the following theorem:

**Theorem 1**. *Cell d*_*ij *_*on diagonal i *- *j with value D dominates all cells d*_*kl *_*in the list with k *<* i*, *i *- *j *<* k *- *l *≤ *n *- *m and values *<*D*, *meaning all list cells in rows above it and columns with larger indices*.

*Proof*. Since horizontal transitions carry a cost of 0, all cells in row *i *and column *l *with *j *<* l *≤*n *- *m *have a score of at most *D*. All cells *d*_*kl *_in the list, residing in diagonals *k *- *l *with *i *- *j *<* k *- *l *≤*n *- *m *and in rows *k *with *k *<* i *lead diagonal transitions to cells *d*_*k *+ 1, *l *+1 _with score at most *D*, since *a*_*l *_≠ *b*_*k *_(or *d*_*kl *_would not belong to the list, dominated by *d*_*k*+1, *l*+1_). This implies that no diagonal transition from these cells can produce a value smaller than *D *in any cell on row *i *and column > *j *via a path passing through these cells, since values in the paths are monotonically increasing (because all edit operations have non-negative costs). If we now examine the vertical transitions emanating the *d*_*kl *_cells under consideration, they also result in paths propagating scores at least *D*, which again cannot result in a better score on the cells on row *i *and column > *j*. All cells on diagonals <*i *- *j *do not need to be considered, since they cannot be reached from the claimed dominated cells of this theorem, unless a path reaches them through a cell in diagonal *i *- *j*. But in corollary 2 we showed that cells on this diagonal with scores ≥ *D *are already dominated by *d*_*ij*_. Thus all *d*_*kl *_cells are dominated by *d*_*ij*_.   □

The next corollary follows from the domination theorem 1:

**Corollary 3**. *No two cells in the list reside on the same column*.

*Proof*. Before a new candidate cell *d*_*ij *_is inserted in the list, any list cell on the same column will be removed, since it is dominated by the newly inserted cell, based on the previous theorem.   □

Now we have the necessary tools to prove the following lemma:

**Lemma 3**. *When iteration D starts, with *1 ≤ *D *≤ *s *- (*m *- *n*), *all cells in the linked list have either a score of D *- 1 *or D *- 2.

*Proof*. Initially, after the initialization, the list holds cells with value 0, so the lemma holds. Every time a cell is inserted in the list it will remain until it is dominated by another cell or the algorithm terminates. Unless a cell with score *D *in the list is dominated and removed before its transitions are examined, when the algorithm reaches that cell the diagonal transition emanated from the cell will produce the next candidate, with score *D *+ 1, to be inserted in the list. The second time this cell is visited, the vertical transition from it will be examined. In that case, the next candidate with score *D *+ 2 will dominate the current cell, according to the previous theorem. Thus, even if a cell is not dominated by another inserted cell, it will be dominated by its siblings.   □

A direct consequence of the previous lemma is the following:

**Corollary 4**. *At most two cells in the list can reside in the same diagonal, and their values differ by *1. *This holds for same row list cells as well*.

A pseudo-code description of the algorithm is presented below. The description excludes special cases requiring substitutions of the currently examined cells of the list and only presents the operations of the algorithm in the part of the matrix left of the main diagonal. The procedure interfacing the left and right linked lists is omitted as well. The algorithm can be studied in more detail from the available code.

Initialize lookahead arrays *X*

Initialize linked list *L*

score *D *:= 0

line *l *:= 0

column *c *:= 0

**while **Not reached main diagonal **do**

   insert *d*_*lx *_:= X[*a*_*l*_][*c*] into *L*

   *c *:= *x*

   *l *+ +

end while

**while **Not reached cell *d*_*mn *_**do**

   D + +

   Current Cell *d*_*ij *_:= *L *→ head

   **repeat**

      **if ***d*_*ij *_= *D *- 1 **then**

         *d*_*ij *_:= *process_next_candidate*(*d*_*i*+1, *j*+1_)

      **else**

         *d*_*ij *_:= *process_next_candidate*(*d*_*i*+1, *j*_)

      **end if**

   **until ***d*_*ij *_= *L *→ head

end while

**Function **process_left_candidate(*d*_*kl*_)

**while ***a*_*l *_= *b*_*k *_**do**

   *k *+ +

   *l *+ +

end while

Insert *d*_*kl *_in list *L*

Remove dominated *d*_*ij *_→ next by *d*_*kl *_from *L*

**while **not reached diagonal of *d*_*ij *_→ next **do**

   *process_left_candidate*(X[*a*_*k*+1_][*l *+ 1])

end while

**return ***d*_*ij *_→ next

### Algorithm complexity

The algorithm described in the previous section is score based and as such the main loop executes an equal number of times with the value recorded at cell *d*_*mn *_of the edit graph. Since we add the value *n *- *m *to that score in order to obtain the edit distance of strings A and B, the total number of iterations is equal to *s *- |*n *- *m*|.

At all times during the execution of the algorithm the linked list contains at most *m *cells, which is a direct consequence of corollary 3. Also, due to corollary 4, there can be at most 2*s *cells in the list at any given time, since the maximum number of diagonals on which the algorithm processes cells is s, consisting of the center of the matrix and diagonal bands of size (*s *- |*n *- *m*|)/2 from each side of the center, accessed while the algorithm iterates. Basically, for every two iterations of the algorithm, one further diagonal from each side of the center of the matrix is accessed.

All cells in the list are accessed in order and without backtracking during each iteration. Each cell undergoes through a constant number of structural accesses, once when it is inserted in the list, once when it is removed and two times when the diagonal and vertical transitions from this cell are examined, if there is a chance before it is dominated. During each iteration there are other cells accessed, the candidates for insertion in the list. While processing these cells we are advancing both the indices of columns and rows without backtracking, which proves, as with list cells, that there are at most *m *or *s *candidate cells examined during each iteration.

A candidate cell may be accessed several times while compared to a list cell, in order to determine a dominance relation. A list cell can also be accessed several times during the same process, to check whether it is dominated. However, the amortized cost for each cell is constant. Every time a candidate cell is re-examined, a cell from the list has been removed. And every time a list cell is re-examined, in the previous step it was not dominated by a candidate cell, the latter then having being inserted in the list and not being examined again on that iteration. Since each time we advance through either a candidate or a list cell, and since both sets have O(min(*m*, *s*)) cells (under the assumption that *m *≤ *n*), the total number of constant time operations during an iteration is O(min(*m, n, s*)).

This analysis demonstrates that the total running time of our algorithm is O((*s *- |*n *- *m*|)·min(*m, n, s*) + *m *+ *n*), where the last linear *m *+ *n *component represents the time necessary to initialize the lookahead tables. It can be easily verified using simple algebra that *s *- |*m *- *n*| ≤ min(*m*, *n*), which provides another less tight upper bound of the worst case time behavior of the algorithm, O(min(*m*, *n*, *s*)^2 ^+ *m *+ *n*). We can therefore observe that the quadratic factor in the time complexity is independent of the longest string being compared. The space usage of this algorithm is O(*m *+ *n*), dominated by the size of the lookahead tables kept in memory. This completes the proof of the next theorem:

**Theorem 2**. *The edit distance s of two strings A and B with lengths n and m respectively can be computed in time O*((*s *- |*n *- *m*|)·min(*m*, *n*, *s*) + *m *+ *n*) *and in space *O(*m *+ *n*).

## Results and Discussion

We have implemented our new algorithm to test its performance in practice. For comparison purposes, we implemented the basic O(*mn*) algorithm, also known as Needleman-Wunsch [3], as well as the Ukkonen O(*s*·min(*m*, *n*)) algorithm [11]. All algorithms were implemented in perl, using the same input/output procedures and no optimizations. Benchmarking was performed with the benchmark perl module for the experiments averaging a large number of random runs, and the *time *unix command for individual experiments, the same method always used across algorithms. All tests were performed on an 8 GB RAM 2.93 GHz Intel processor IBM compatible desktop machine, running ubuntu linux. In all test cases the data completely fit in the main memory.

Since perl does not support pointer structures efficiently, we implemented the double linked list with arrays, using the fact that no two cells in the list can reside on the same column. This way we access list cells using their column index. As such, the list occupies more space than the minimum possible, where the implementation may have been more efficient in another programming language supporting these structures.

Ukkonen's algorithm implementation was based on the outline found in [11] and the more detailed description found in [13]. The version used is particularly simple by making use of recursion, but has larger than linear space demands, specifically O(*s*·max(*m*, *n*)). The basic algorithm was implemented using linear space and row-by-row iterations.

**Figure 4:**
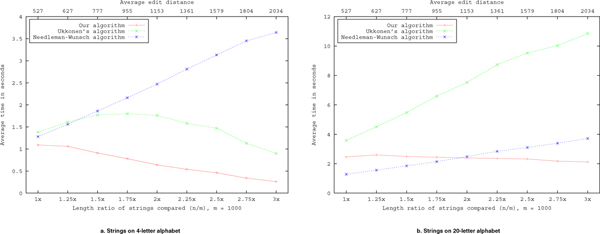
**Performance on random strings**. Edit distance calculations on random strings with different length ratios, comparing the performance of ours, Ukkonen's and the basic algorithms.

The first two experiments were run on random sequences over alphabets of 4 and 20 characters respectively, similar to random DNA/RNA and amino acid sequences. The length of the first sequence from the two compared was set at 1000 characters, where the length of the second sequence varied between 1000 and 3000 characters. We examined a total of nine length ratios *n*/*m *values between 1 and 3 (1 ≤ *n*/*m *≤ 3). For each length ratio, 100 different comparisons were run, with the execution time and edit distance values averaged among these. The results are depicted in Fig. 4.

In these simulations it is worth noticing significant performance improvement of the new algorithm with increasing length ratio of the random strings, although the total length of the strings is increasing. This is not surprising, since the number of iterations *s *- |*m *- *n*| is decreasing, caused by a slower increase in edit distance than difference between the lengths of the two strings.

Ukkonen's algorithm performs poorly when comparing random strings over a large alphabet, because of the large expected edit distance value in these cases. This algorithm is designed for comparing similar strings, which is the case most often encountered in practice. In contrast, the basic algorithm, owing to its simplicity, performs uniformly and surpasses the other algorithms when the edit distance is large compared to string length, unless when the *s *- |*n *- *m*| value becomes small enough, where our algorithm takes the lead.

Next, we designed computational experiments performing comparisons most often encountered in practice, drawn from the computational biology domain. In all examples the sequence pairs examined have comparable lengths, not differing more than 5%. The results are presented in Table 1. The first simulation involved 1000 random sequence pair comparisons from a pool of approximately 6800 vetted 16S ribosomal RNA sequences, provided by the Ribosomal Database Project (RDP). [14] These sequences average about 1350 characters in size, drawn from an alphabet of size 4. A random pair of 16S rRNA sequences from the same genus and another from the same class but different order are compared in the next two lines, followed by a comparison of two viral genomes and two virion proteins. As these results demonstrate, the performance of our algorithm compares favorably to Ukkonen's algorithm, which is asymptotically slower but has smaller constants, while the basic algorithm is outperformed in almost every case, except when matches are sparse. Performance comes with some cost though and it is interesting to note that the size of the program implementations of the three algorithms, the basic, Ukkonen's and ours, is 80, 160 and 700 lines of code respectively.

**Table 1:** Algorithm performance comparing biologically related sequences of similar length

Sequence A	Sequence B	Alphabet size	(Average) length	Our algorithm (sec)	Ukkonen's algorithm (sec)	Basic algorithm (sec)	(Average) edit distance
Random 16S rRNA sequence	Random 16S rRNA sequence	4	1350	0.679	0.811	2.554	421.3

Hyphomonas 16S rRNA (AF082798)	Hyphomonas 16S rRNA (AF082795)	4	1330	0.25	0.18	2.14	46

Alphaproteobacteria 16S rRNA (AJ238567)	Betaproteobacteria 16S rRNA (AJ239278)	4	1320	0.42	0.46	2.07	318

Cucumber necrosis virus genome	Lisianthus necrosis virus genome	4	4790	6.70	6.32	28.27	1154

Human poliovirus 1 virion protein	Human Rhinovirus A virion protein	20	870	1.02	1.05	0.88	472

The perl implementations of all three algorithms used in this paper for performance comparisons can be downloaded online. [15]

## Conclusion

In this paper we have provided the design, analysis and implementation of a new algorithm for calculating the edit distance of two strings. This algorithm is shown to have improved asymptotic time behavior, while it is also demonstrated to perform very well in practice, especially when the lengths of the strings compared differ significantly. The performance of our algorithm in this case, which is encountered less often in instances of the edit distance problem, could find application in the related Longest Common Subsequence (LCS) and other similar problems solved with dynamic programming techniques.

Future directions for this algorithm include the investigation of further practical applications of the techniques described to other similar problems, as well as generalizing the results to cover additional edit operations such as swaps.

## Competing interests

The authors declare that they have no competing interests.

## Authors' contributions

Both authors designed and analyzed the algorithm, DP implemented the algorithm, performed the experiments and wrote the manuscript. Both authors read and approved the final manuscript.
